# Community structure of the metabolically active rumen bacterial and archaeal communities of dairy cows over the transition period

**DOI:** 10.1371/journal.pone.0187858

**Published:** 2017-11-08

**Authors:** Zhigang Zhu, Samantha Joan Noel, Gareth Frank Difford, Waleed Abu Al-Soud, Asker Brejnrod, Søren Johannes Sørensen, Jan Lassen, Peter Løvendahl, Ole Højberg

**Affiliations:** 1 Department of Animal Science, Aarhus University, Tjele, Denmark; 2 Center for Quantitative Genetics and Genomics, Department of Molecular Biology and Genetics, Aarhus University, Tjele, Denmark; 3 Department of Biology, Faculty of Science, University of Copenhagen, Copenhagen, Denmark; University of Wisconsin Madison, UNITED STATES

## Abstract

Dairy cows experience dramatic changes in host physiology from gestation to lactation period and dietary switch from high-forage prepartum diet to high-concentrate postpartum diet over the transition period (parturition +/- three weeks). Understanding the community structure and activity of the rumen microbiota and its associative patterns over the transition period may provide insight for e.g. improving animal health and production. In the present study, rumen samples from ten primiparous Holstein dairy cows were collected over seven weeks spanning the transition period. Total RNA was extracted from the rumen samples and cDNA thereof was subsequently used for characterizing the metabolically active bacterial (16S rRNA transcript amplicon sequencing) and archaeal (qPCR, T-RFLP and *mcrA* and 16S rRNA transcript amplicon sequencing) communities. The metabolically active bacterial community was dominated by three phyla, showing significant changes in relative abundance range over the transition period: *Firmicutes* (from prepartum 57% to postpartum 35%), *Bacteroidetes* (from prepartum 22% to postpartum 18%) and *Proteobacteria* (from prepartum 7% to postpartum 32%). For the archaea, qPCR analysis of 16S rRNA transcript number, revealed a significant prepartum to postpartum increase in *Methanobacteriales*, in accordance with an observed increase (from prepartum 80% to postpartum 89%) in relative abundance of 16S rRNA transcript amplicons allocated to this order. On the other hand, a significant prepartum to postpartum decrease (from 15% to 2%) was observed in relative abundance of *Methanomassiliicoccales* 16S rRNA transcripts. In contrast to qPCR analysis of the 16S rRNA transcripts, quantification of *mcrA* transcripts revealed no change in total abundance of metabolically active methanogens over the transition period. According to T-RFLP analysis of the *mcrA* transcripts, two *Methanobacteriales* genera, *Methanobrevibacter* and *Methanosphaera* (represented by the T-RFs 39 and 267 bp), represented more than 70% of the metabolically active methanogens, showing no significant changes over the transition period; minor T-RFs, likely to represent members of the order *Methanomassiliicoccales* and with a relative abundance below 5% in total, decreased significantly over the transition period. In accordance with the T-RFLP analysis, the *mcrA* transcript amplicon sequencing revealed *Methanobacteriales* to cover 99% of the total reads, dominated by the genera *Methanobrevibacter* (75%) and *Methanosphaera* (24%), whereas the *Methanomassiliicoccales* order covered only 0.2% of the total reads. In conclusion, the present study showed that the structure of the metabolically active bacterial and archaeal rumen communities changed over the transition period, likely in response to the dramatic changes in physiology and nutritional factors like dry matter intake and feed composition. It should be noted however that for the methanogens, the observed community changes were influenced by the analyzed gene (*mcrA* or 16S rRNA).

## Introduction

The peri-parturition transition phase of dairy cows is characterized by dramatic changes in host physiology and nutrient metabolism, imposing great challenges to the animals [1], and different feeding strategies have been explored to reduce e.g. the incidence of rumen acidosis in the immediate postpartum period [2]. DNA-based studies have reported changes of the dairy cow rumen microbiota over the transition period and the correlations between specific members of the rumen microbiota and e.g. milk phenotype and rumen VFA profile[3–5]. However, there is still a lack of RNA-based studies investigating the dynamics of the metabolically active members of the rumen microbiota over the transition period.

It is anticipated that there is a positive correlation between ribosomal RNA (rRNA) content and metabolic activity of microbial cells[6,7]. A comparison between DNA (16S rRNA gene) and RNA (16S rRNA) clone libraries revealed distinct phylotypes of a marine bacterial community for the two approaches[8], thus indicating that the most abundant members of a community may not necessarily be the most active or vice versa. The majority of published molecular investigations on the rumen microbiota are DNA-based indicating that there is still limited knowledge about the structure and function of the metabolically active rumen microbiota. Kang et al (2013) reported, however, certain *Proteobacteria* and uncultured methanogens to be more dominating in RNA-based than in DNA-based ribosomal 16S analyses of the rumen microbiota of cows fed grain-based diets[9]. Analyzed by qPCR, increasing proportions of dietary corn silage showed no effect on dairy cow rumen microbiota at the DNA level, however at the RNA level, higher amounts of *Prevotella* transcripts were observed for cows fed high corn silage rations [10]. The metabolically active rumen microbiota of dairy cows has further been addressed in studies focusing on diet-induced alterations [10,11] and the successional colonization of fresh perennial ryegrass in the rumen[12]. The rumen microbiota of beef steers, analyzed by total RNA-sequencing as well as 16S rRNA and rRNA gene amplicon-sequencing, revealed unique microbial taxa specific to each approach, where e.g. the bacterial families *Desulfovibrionaceae*, *Elusimicrobiaceae*, and *Sphaerochaetaceae* were detected only with total RNA and 16S rRNA amplicon sequencing, but not with 16S rRNA gene amplicon sequencing[13].

The rumen archaeal community has received special attention in particular with respect to the methanogens being a major source of anthropogenic methane emissions. DNA-based studies have enhanced our understanding of the rumen microbial ecology of the methanogenic archaea residing in different animal species[14–16], however, according to a RNA-based study, an uncultivated methanogen clade contributed one-third of RNA-derived methyl coenzyme-M reductase subunit A (*mcrA*) sequences, whereas the clade was not observed in DNA-derived sequences[9]. Furthermore, another RNA-based work (metatranscriptomics) found significant differences in the expression of methanogenesis pathway genes between high methane yield and low methane yield sheep, whereas DNA-based analysis showed no difference between the two[17]. Finally, a metatranscriptomic investigation showed that transcripts (16S rRNA and methyl coenzyme M reductase) of the poorly characterized *Thermoplasmata* (*Methanomassiliicoccales*) archaea were reduced upon dietary supplementation with rapeseed oil in lactating cows, whereas *Methanobacteriales* transcripts were unaffected by the dietary treatment[18]. Thus, RNA-based analyses may have different perspectives from DNA-based analysis when evaluating the rumen archaeal community.

In a parallel study, we investigated the community composition of rumen *Bacteria* and methanogenic *Archaea* of dairy cows over the transition period by DNA based analyses (manuscript in preparation). The aim of the present study was to investigate the community composition of the metabolically active rumen *Bacteria* and *Archaea* over the transition period in dairy cows by RNA (cDNA) based techniques. This was done using high throughput sequencing (Illumina MiSeq) of transcript amplicons, using universal prokaryotic 16S rRNA primers for targeting *Bacteria* and *Archaea* as well as *mcrA* primers for targeting methanogenic *Archaea*, specifically. The methanogenic *Archaea* were further analyzed by *mcrA*-targeted T-RFLP analysis and quantification (qPCR) of total methanogenic archaea (*mcrA* gene transcript numbers) and two major methanogen orders, *Methanobacteriales* and *Methanomassiliicoccales* (16S rRNA gene transcript numbers). To our knowledge, this is the first study reporting the dynamics of the rumen metabolically active bacterial and archaeal communities in dairy cows over the transition period.

## Materials and methods

### Animals, diets and rumen sampling

The animal experimental protocol was approved by The Animal Experiments Inspectorate, Danish Veterinary and Food Administration, Ministry of Environment and Food of Denmark (Approval number 2016-15-0201-00959). Ten primiparous Holstein cows with a close predicted calving date were maintained in the same rearing environment at a research farm (Danish Cattle Research Centre; www.DKC-Foulum.dk). Before calving, all the cows were housed together in a barn with straw padding and fed *ad libitum* with a low grain pre-partum diet (S1 Table). After calving, they were transferred to a barn equipped with an automatic milking robot (VMS, DeLaval, Tumba, Sweden) for lactating cows where they were fed *ad libitum* with a high-grain post-partum diet and a limited amount of concentrate was delivered while milking. Individual dry matter intake was recorded over throughout the experimental period (S1 Fig). All cows always had free access to drinking water. Rumen samples from each cow were collected in the morning between 9 am and 10 am orally with a flora scoop (Guelph, Canada)[19]. To minimize rumen-sampling variation, the same person collected all samples, following an outlined procedure [19], recognizing however that the location of the flora scoop may still have differed somewhat from sampling to sampling. The cows were sampled once a week for seven consecutive weeks spanning the transition period. Approximately 40 mL rumen sample (rumen fluid mixed with fine particles) were withdrawn and poured into a 50 mL polypropylene centrifuge tube and transferred on ice to the laboratory. A homogeneous subsample containing rumen fluid and fine feed particles of 1.2 mL was snap frozen in liquid nitrogen, and stored at -80°C until further analysis.

### RNA extraction and cDNA synthesis

Snap frozen rumen samples were thawed at room temperature and 250 *μ*L of homogenously mixed subsample were applied for RNA extraction according to a standard phenol-chloroform bead-beating procedure originally published by Paulin et al.[20]. Raw RNA extracts were purified with NucleoSpin RNA clean up XS kit according to the manufacturer’s instructions (Macherey-Nagel, Duren, Germany) and 2 *μ*L of Riboblock RNase inhibitor (Thermo Fisher Scientific, MA, USA) was added to the purified RNA sample (30 *μ*L) to minimize RNA degradation. Genomic DNA was removed from the RNA extracts using DNase I kit (Thermo Fisher Scientific, MA, USA) according to manufacturer’s instructions. The removal of all contaminating DNA was confirmed by PCR amplification of the RNA extracts with universal bacterial 16S rRNA gene primer (Forward 5’- CGG YCC AGA CTC CTA CGG; Reverse 5’- TTA CCG CGG CTG CTG GCA C) resulting in no product being formed. The purification of DNase treated RNA was performed with the MEGAclear kit (Life Technologies, Carlsbad, CA) according to manufacturer’s instructions. Total RNA extracts were quantified by using Qubit RNA broad range (20-1000ng) assay kit (Life Technologies, Carlsbad, CA). The reverse-transcription of RNA (10 *μ*L) to single-strand cDNA was conducted by using the High-capacity cDNA reverse transcription kit (Life Technologies, Carlsbad, CA) according to manufacturer’s instructions and synthesized cDNA template was stored at—20°C until further analysis.

### 16S rRNA transcript amplicon sequencing

The cDNA template was adjusted to a concentration of 10 ng/*μ*L using Rnase-free water. Universal prokaryotic primers 341F (5’-CCT AYG GGR BGC ASCAG) [21]and modified 806R (5’-GGA CTA CNN GGG TAT CTA AT)[22], were used to amplify the V3-V4 region of 16S rRNA gene. The PCR mixture consisted of 1 × Accuprime^™^ PCR Buffer II (15 mM MgCI_2_), 0.3 U AccuPrime^™^ Taq DNA polymerase, 0.5 *μ*M of each primer (10 pmol/ *μ*L), 2 *μ*L diluted template and water to a total of 20 *μ*L (Life Technologies, Carlsbad, USA). The PCR incubation conditions consisted of an initial activation of the hot-start polymerase at 94°C for 2 min, followed by 30 cycles of 94°C for 20s, 56°C for 20s and 68°C for 30s, and final extension at 68°C for 5 min. The PCR products (20 *μ*L) were purified with 15 *μ*L AMPure XP Beads (Beckman Coulter, Inc.). The purified PCR products were checked to be of the expected size on an agarose gel and then diluted to equimolar concentration (Approx. 5 ng/*μ*L). Indexes and adaptors were attached to the PCR products with Nextera DNA library preparation kit (Illumina, San Diego, CA) through a second PCR step. The PCR products were purified again with AMPure XP Bead (Beckman Coulter, Inc.) and equal-molar concentration of PCR product was pooled and sequenced as 250 bp paired-end reads using Illumina MiSeq platform (Illumina, San Diego, CA). Raw sequence files were deposited in NCBI Sequence Read Archive (SRA) under accession No. SRP082151.

### 16S rRNA transcript amplicon sequencing data analysis

An average number of 68,806 reads were generated for this study. The sequence data analysis was performed with the LotuS (Less operational taxonomic units Scripts) pipeline[23]. Within LotuS the demultiplexing and quality-filtering of sequences were carried out with *sdm* options, where strict filtered high-quality reads (minimum sequence length of 230 bp and minimum average quality score of 27) were used for OTU clustering while less strict filtered mid-quality reads (minimum length of 230 bp and minimum average quality score of 20) were used for estimating OTU abundance. Sequences with homo-nucleotide numbers over 8 were discarded. Filtered reads were clustered into OTUs using UPARSE pipeline [24] and a representative sequence from each OTU was assigned taxonomy using RDP classifier[25], according to SILVA 16S rDNA database[26]. From the aligned sequences, a phylogenetic tree was constructed using the gamma model of sequence evolution in FastTree2[27]. The output OTU table (OTU.biom) file and phylogenetic tree (Tree.tre) file from LotuS pipeline were used as input for the QIIME pipeline (version 1.9.0) [28]to calculate alpha diversity indexes, where the richness (Chao1 index), the number of distinct OTUs (Observed species) and the phylogenetic diversity (PD whole tree) were estimated. To get an overview of the shifts of rumen metabolically active bacterial community, principal coordinate analysis (PCoA) was implemented on the weighted UniFrac distance matrix as suggested by Hamady and Knight[29]. The statistical significance of weekly-based sample groups was tested on the weighted UniFrac distance matrix using the ANOSIM method[30].

### Quantification of total methanogen and two major methanogen orders by qPCR

For the quantifications of total methanogenic archaea and two major orders of methanogens (*Methanobacteriales* and *Methanomassiliicoccales*), *mcrA* gene primer and order specific 16S rRNA gene primers were used for qPCR amplification of cDNA (S2 Table). Triplicates of each sample were prepared and all PCR reactions were performed on a 384-well plate and run on a ViiA 7 real-time PCR system (Thermo Fisher Scientific, Waltham, MA). Each PCR reaction contained a total volume of 10 *μ*L PCR mixture, including 5 *μ*L of iTaqTM Universal SYBR Green Supermix (Bio-rad, CA,USA), 1 *μ*L of each forward and reverse primer (10 pmol *μ*L^-1^), 2.5 *μ*L of ddH_2_O and 0.5 *μ*L of cDNA template. The amplification condition consisted of: denaturation at 95°C for 25 s followed by 40 cycles of denaturation at 95°C for 15 s, annealing at 60°C for 35 s and extension at 72°C for 30 s. Total methanogen abundance was quantified by *mcrA* gene specific primer and a standard curve was made by ten-fold serial dilutions of DNA extracts from *Methanomassiliicoccus luminyensis* pure culture purchased from Leibniz-institute DSMZ GmbH (Braunschweig, Germany). To quantify the 16S rRNA copy number of two major methanogen orders, *Methanobacteriales* and *Methanomassiliicoccales*, individual standard curves were made by ten-fold serial dilutions of DNA extracts from *Methanobrevibacter ruminantium* and *Methanomassiliicoccus luminyensis* pure cultures, respectively, which were purchased from Leibniz-institute DSMZ GmbH (Braunschweig, Germany). Standard curves with an amplification efficiency between 85% and 100% and R square value greater than 0.99 were accepted for subsequent calculation of transcripts numbers.

### T-RFLP analysis of *mcrA* transcripts

For amplifying the cDNA templates, *mcrA* gene specific fluorescently labeled primers (forward primer 5’-GGT GGT GTM GGD TTY ACH CAR TA modified from Steinberg and Regan et al. (2008) [31]and reverse primer 5’-FAM CGT TCA TBG CGT AGT TVG GRT AGT) was used. Four replicates of each sample were prepared and each PCR reaction contained a total volume of 20 *μ*L, including 2 *μ*L 10× AccuPrimeTM PCR buffer II (Invitrogen, CA, USA), 1 *μ*L of each primer (10 μM), 0.12 *μ*L Taq DNA polymerase, 13.88 *μ*L of water and 2 *μ*L of cDNA templates. The PCR amplification procedure consisted of: an initial activation at 94°C for 2 min, followed by 35 cycles of denaturation at 94°C for 20 s, annealing at 56°C for 20 s and extension at 68°C for 30 s, and final extension at 68°C for 5 min. Pooled PCR products for each sample were purified using QIAquick PCR purification kit (Qiagen GmbH, Hilden, Germany) following the manufacturer’s instructions. Purified PCR amplicons were digested using endonuclease *Taq*I (New England Biolabs, Ipswich, MA). Digested PCR fragments were precipitated and mixed with 0.2 *μ*L Megabase ET900-R size standard (GE Healthcare, Buckinghamshire, UK) and quantified on an ABI 3730XL Capillary Sequencer (Life Technologies, Carlsbad, CA).

### *mcrA* transcript amplicon sequencing

Sequencing library was constructed from cDNA templates by using a two-step PCR procedure targeting the *mcrA* transcripts. The first PCR reaction was conducted in triplicates for each sample; the preparation of PCR mixture and the PCR reaction condition were identical to the aforementioned T-RFLP procedure. The PCR products were purified with AMPure XP Beads (Beckman Coulter, Inc.) and then pooled PCR products for each sample were processed according to a standard Illumina MiSeq protocol (Illumina, San Diego, CA). Briefly, amplicon primers with overhang (Forward overhang: 5’ TCGTCGGCAGCGTCAGATGTGTATAAGAGACAG-GGTGGT GTMGGDTTYACHCARTA; Reverse overhang: 5’ GTCTCGTGGGCTCGGAGATGTGTATAAGAGACAG-CGTTCATBGCGTAGT TVGGRTAGT) were used to amplify the *mcrA* amplicons from the first PCR run, followed by the purification of PCR products with AMPure XP Beads (Beckman Coulter, Inc.) and subsequently, the addition of dual indices and Illumina sequencing adapters with a Nextera DNA library preparation kit (Illumina, San Diego, CA). Equimolar concentrations of individual samples were pooled and sequenced on Illumina MiSeq platform (Illumina, San Diego, CA) using the 250 bp paired end protocol.

### *mcrA* transcript amplicon sequencing data analysis

A total of 1,139,525 sequences were generated from 70 samples. After the removal of primers and adaptors (Cutadapt version 1.9.1), paired end reads were merged by USEARCH[32]. The merged reads with an average read length of 424 bp were quality filtered based on a maximum expected error of 1.0. Further analysis was performed on QIIME platform (QIIME 1.9.1) [28]with a combined sequence file from all the samples. A custom database was constructed by downloading all *mcrA* gene sequences from the FunGene Pipeline version 8.1[33]. OTUs were generated from the combined sequence file using the (*pick_otus*.*py*) script in QIIME with UCLUST OTU picking method[32], and a cut-off value of 84% was applied for species level, as recommended by Yang et al. (2014)[34]. The taxonomy assignment of representative sequences from each OTU was performed with the *assign_taxonomy*.*py* script using default method against our custom *mcrA* gene sequences collection. For those sequences without clear taxonomic assignments, a further blasting against the NCBI nucleotide database was performed and the top hits with an identity value greater than 85% were retained. Thereafter, taxonomic information from the blast analysis was added to the OTU-table for the calculations of alpha diversity (Chao1, Observed species and PD whole tree) and beta diversity, for which principal coordinate analysis (PCoA) was performed on the weighted UniFrac distance matrix[29]. The statistical significance of weekly-based sample groups was tested on the weighted UniFrac distance matrix using the ANOSIM method[30].

### Phylogeny of dominant methanogen OTUs

To visualize the phylogeny of dominant OTUs, with a total sequence number great than 5956 across all samples, representative *mcrA* sequences from 22 *Methanobacteriales*-related OTUs and 14 *Methanomassiliicoccales*-related OTUs were picked out, and a phylogenetic tree was constructed with maximum likelihood method in MEGA6[35].

### Statistical analysis

The seventy samples (7 from each of 10 cows) were grouped into weeks relative to their actual calving date, leading to seven sample groups; three weeks before parturition (w-3), two weeks before parturition (w-2), one week before parturition (w-1), the first week after parturition (w1), the second week after parturition (w2), the third week after parturition (w3) and the fourth week after parturition (w4). Consequently, these seven groups from w-3 to w4 consisted of 10, 9, 10, 10, 9, 9 and 13 samples, respectively. Since animals were sampled multiple times and the individual animals were sampled in different sample weeks, the assumption of independence of residuals within animals and between sample weeks was tested by fitting linear mixed models using PROC MIX command in SAS (SAS 9.3, SAS Institute Inc.). Fixed effects were assessed by F tests and random models by log-likelihood ratio tests. The model that fit the data best, included “Weeks relative to parturition” as a fixed effect, and animals and sample weeks were considered as random effects. The relative abundance of bacteria taxa was calculated as the sequence number in a specific OTU divided by the total sequence numbers detected in an individual sample. Similarly, the relative abundance of T-RFs was calculated as the peak height of individual T-RFs divided by the total peak heights within each sample. The relative abundance of microbial taxa and T-RFs were considered as dependent variables. When the fixed effect “Weeks relative to parturition” was significant (*P*-value ≤ 0.05), a further least square means t-test was carried out to compare the seven weekly groups and turkey’s was used as a multiple comparison adjustment of the *P*-values. *P*-values ≤ 0.05 were considered as significant and *P*-values between 0.05 and 0.10 represented a trend.

## Results

### Bacterial 16S rRNA transcript amplicon profile

**Fig 1:**
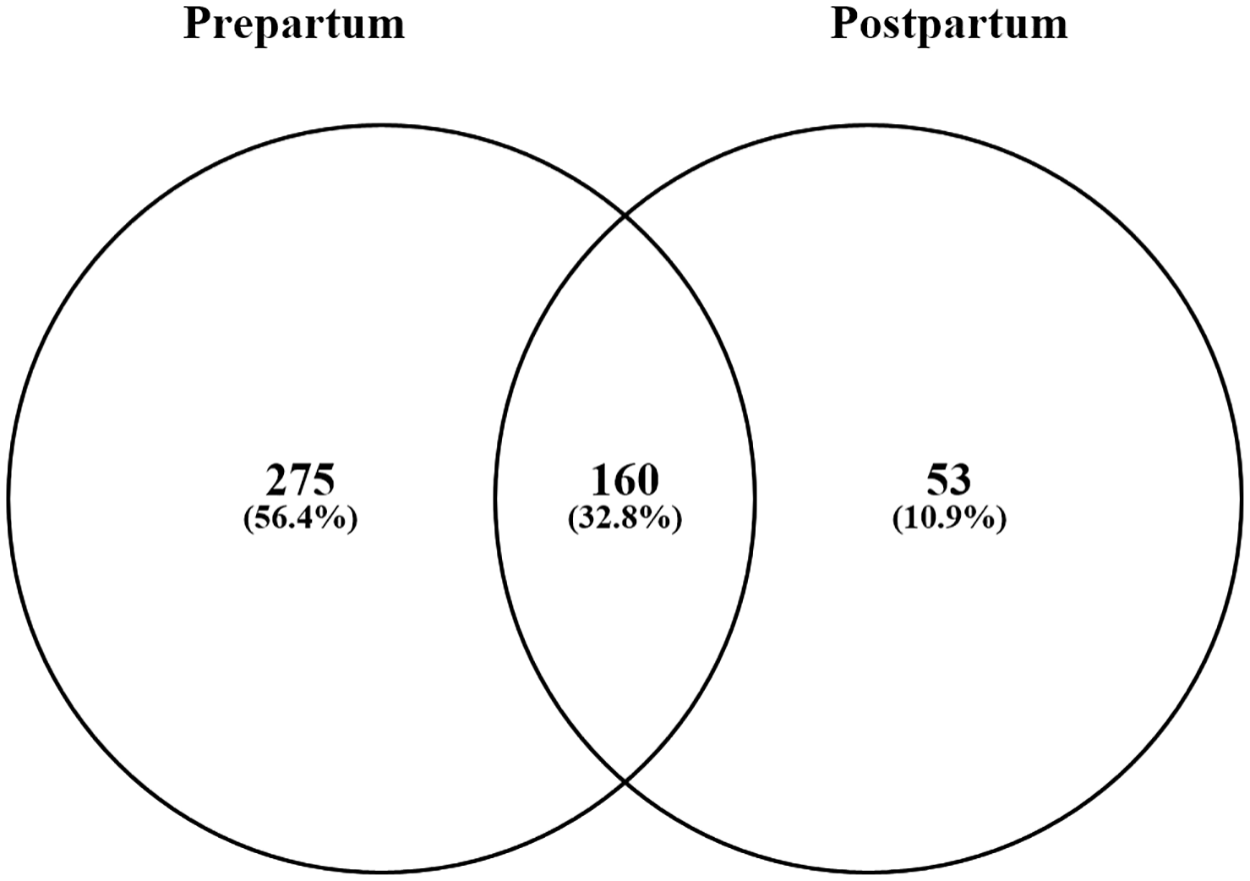
Distribution (Venn diagram) of OTUs among the 16S rRNA transcript amplicons. The OTUs identified in the 16S rRNA transcript amplicon library of all the prepartum (435) and postpartum samples (213) are included in the Venn diagram and grouped as OTUs either unique to or shared between the prepartum and postpartum microbiome.

A total of 5,647 OTUs were identified from the bacterial 16S rRNA transcript profile. There were 435 OTUs ubiquitously present in all the prepartum samples, 213 OTUs ubiquitously present in all the postpartum samples and 160 OTUs shared between the prepartum and postpartum samples (Fig 1). Of the 275 OTUs specific to the prepartum microbiome, they were taxonomically associated with *Ruminococcaceae* (76 OTUs), *Prevotellaceae* (23 OTUs), *Lachnospiraceae* (21 OTUs), unclassified *Clostridiales* (16 OTUs) and *Bacteroidales* (12 OTUs). Meanwhile, of the 53 OTUs specific to the postpartum microbiome, they were taxonomically associated with *Lachnospiraceae* (20 OTUs), *Prevotellaceae* (10 OTUs) and *Ruminococcaceae* (4 OTUs). Across the transition period, predominant genera of the metabolically active rumen bacterial community consisted of unclassified *Ruminococcaceae* (22.3%) and *Lachnospiraceae* (7%), *Ruminococcus* (6.9%), *Prevotella* (6.6%) and *Ruminobacter* (3.2%).

**Fig 2:**
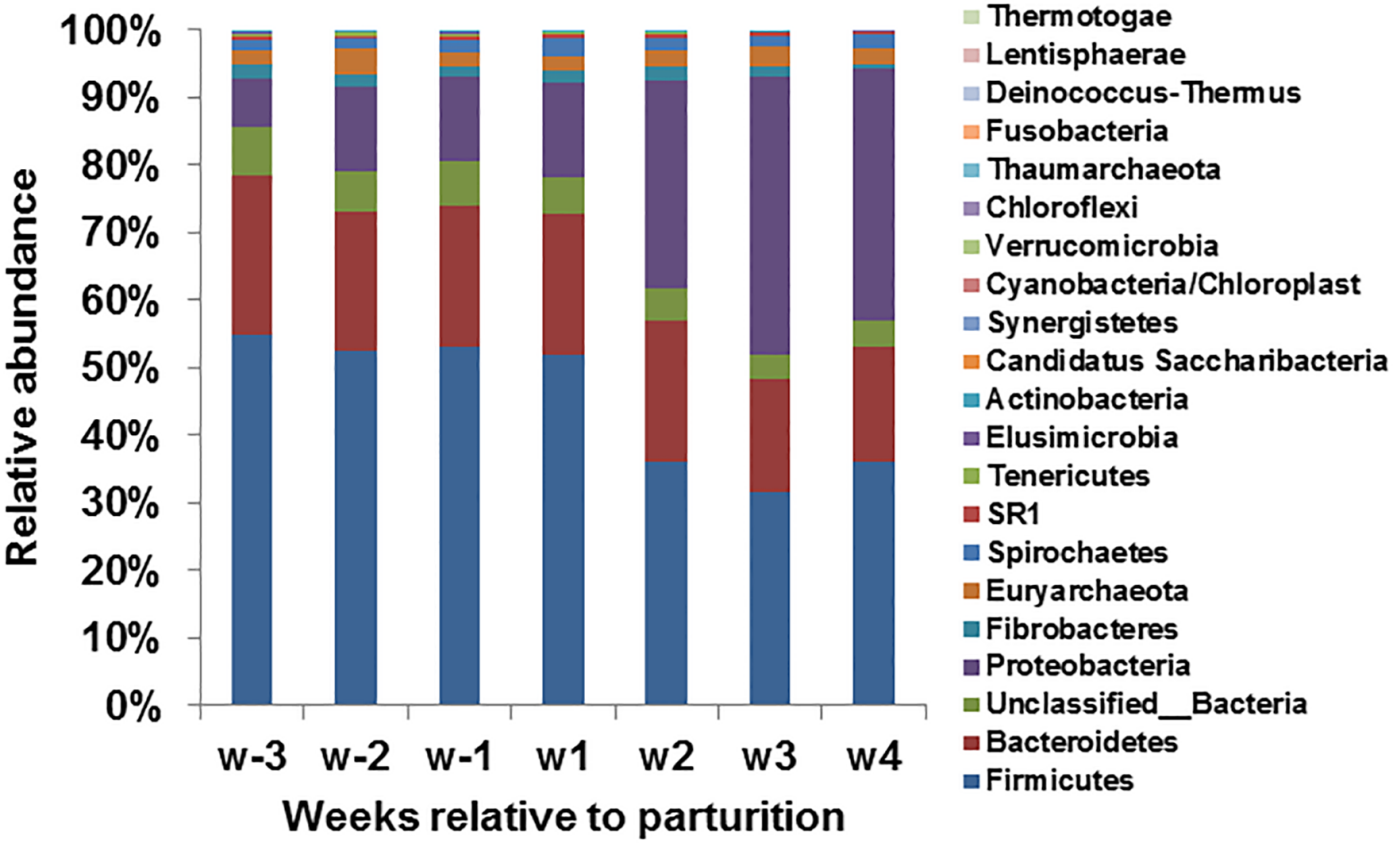
Phylum level composition of the active bacterial and archaeal communities. Relative abundance (percent reads out of total reads) of eighteen bacterial phyla and two archaeal phyla identified among the 16S rRNA transcript amplicons. Bars represent Weekly based sample groups.

The active bacterial community in the rumen consisted of twenty phyla, of which *Firmicutes* (46.91%), *Bacteroidetes* (19.97%), *Proteobacteria* (15.97%), *Spirochaetes* (1.58%) and *Fibrobacteres* (1.52%), were the most abundant five phyla with a median relative abundance value shown in brackets (Fig 2). *Firmicutes* was the most abundant phylum, showing a significant decrease in relative abundance from ~57% in the prepartum period to ~35% in the postpartum period, as did the dominant *Clostridia* class and the *Clostridiales* order of this phylum (Table 1). As the most abundant family of the *Firmicutes* phylum and the *Clostridiales* order, *Ruminococcaceae* showed a significant decrease from the highest relative abundance of ~44% in the prepartum period to the lowest relative abundance of ~22% in the postpartum period. The *Ruminococcaceae* family was further classified into five genera, including *Ruminococcus* (5.81%-7.96%), *Clostridium IV* (0.41%-2.92%), *Saccharofermentans* (0.80%-1.17%), *Flavonifractor* (0.11%-0.21%) and a large group of unclassified *Ruminococcaceae* (13.29%-31.63%). *Clostridium IV* and unclassified *Ruminococcaceae* showed significant decreases in relative abundance from 2.92% to 0.41%, and 31.63% to 13.29% respectively, whereas the remaining three genera showed no significant changes over the transition period. *Lachnospiraceae* (7.24%-10.34%) was another dominant family of the *Firmicutes* phylum, along with unclassified *Lachnospiraceae* (5.69%-8.23%), showing significant increases in relative abundance over the transition period; five dominant genera of this family, including *Butyrivibrio*, *Lachnospiraceae_incertae_sedis*, *Blautia*, *Pseudobutyrivibrio*, and *Moryella*, were low in relative abundance (less than 1%) and showed no significant changes over the transition period. A large group of unclassified *Lachnospiraceae* significantly increased from the lowest relative abundance of 5.69% in the prepartum period to the highest relative abundance of 8.23% in the postpartum period.

**Table 1:** Relative abundance^1^ (%) of major bacterial and archaeal taxa in the rumen of dairy cows across the transition period.

Taxonomic level^3^						Time period relative to parturition^2^							SEM	*P*-value^4^
K	P	C	O	F	G	W-3	W-2	W-1	W1	W2	W3	W4		
*Archaea*														
	*Euryarchaeota*					2.63	3.94	2.53	2.20	2.46	2.24	2.37	0.009	0.75
		*Methanobacteria*				2.09	3.19	2.14	1.82	2.15	1.98	2.12	0.007	0.80
			*Methanobacteriales*			2.09	3.19	2.14	1.82	2.15	1.98	2.12	0.007	0.80
				*Methanobacteriaceae*		2.09	3.19	2.14	1.82	2.15	1.98	2.12	0.007	0.80
					*Methanobrevibacter*	1.81	2.67	1.74	1.36	1.63	1.56	1.69	0.007	0.80
					*Methanosphaera*	0.18	0.29	0.25	0.33	0.43	0.34	0.35	0.001	0.12
					Unclassified*_Methanobacteriaceae*	0.10	0.23	0.15	0.13	0.09	0.08	0.08	0.001	0.47
		*Thermoplasmata*				0.44	0.33	0.22	0.16	0.08	0.07	0.08	0.002	0.22
			*Thermoplasmatales*			0.44	0.33	0.22	0.16	0.08	0.07	0.08	0.001	0.22
				Unclassified*_Thermoplasmatales*		0.43	0.30	0.21	0.14	0.07	0.06	0.08	0.001	0.18
		Unclassified*_Euryarchaeota*				0.10	0.42	0.17	0.22	0.22	0.18	0.17	0.002	0.52
*Bacteria*														
	*Firmicutes*					57.0^a^	53.1^a^^b^	45.1^b^^c^	47.6^a^^b^^c^	35.4^c^	38.6^c^	42.8^a^^b^^c^	0.038	< 0.01
		*Clostridia*				54.8^a^	50.9^a^^b^	42.9^b^^c^	45.6^a^^b^^c^	33.6^c^	36.2^c^	40.8^a^^b^^c^	0.035	< 0.01
			*Clostridiales*			54.3^a^	50.5^a^^b^	42.6^b^^c^	45.4^a^^b^^c^	33.3^c^	35.9^c^	40.5^a^^b^^c^	0.036	< 0.01
				*Ruminococcaceae*		43.7^a^	39.5^a^^b^	32.7^b^^c^	33.2^b^^c^	22.1^c^	22.8^c^	28.9^b^^c^	0.035	< 0.001
					*Ruminococcus*	7.82	6.82	5.81	6.17	6.07	7.96	7.20	0.007	0.16
					*Clostridium IV*	2.92^a^	2.33^a^^b^	2.45^a^^b^	1.84^a^^b^^c^	0.61^c^	0.41^c^	1.17^b^^c^	0.004	< 0.01
					*Saccharofermentans*	1.17	0.96	0.80	0.88	0.68	0.90	0.86	0.001	0.12
					*Flavonifractor*	0.17	0.11	0.13	0.20	0.15	0.21	0.16	0.000	0.35
					Unclassified*_Ruminococcaceae*	31.6^a^	29.3^a^^b^	23.5^b^^c^	24.1^b^^c^	14.6^c^	13.3^c^	19.6^b^^c^	0.031	< 0.0001
				*Lachnospiraceae*		7.36^b^	8.06^a^^b^	7.24^b^	9.70^a^	9.12^a^^b^	10.3^a^	9.10^a^^b^	0.006	< 0.01
					*Butyrivibrio*	0.54	0.54	0.58	0.66	0.59	0.84	0.60	0.001	0.26
					*Lachnospiraceae_incertae_sedis*	0.31	0.50	0.37	0.39	0.39	0.43	0.52	0.001	0.42
					*Blautia*	0.23	0.28	0.28	0.39	0.37	0.32	0.29	0.000	0.03
					*Pseudobutyrivibrio*	0.19	0.22	0.16	0.17	0.16	0.20	0.17	0.000	0.48
					*Moryella*	0.06	0.06	0.05	0.05	0.04	0.07	0.06	0.000	0.57
					Unclassified*_Lachnospiraceae*	5.99^b^	6.42^a^^b^	5.69^b^	7.90^a^	7.43^a^^b^	8.23^a^	7.32^a^^b^	0.005	< 0.01
				Unclassified*_Clostridiales*		3.17^a^	2.88^a^^b^	2.56^a^^b^^c^	2.39^b^^c^	1.96^c^	2.05^c^	2.26^c^	0.002	< 0.01
		Unclassified*_Firmicutes*				1.96	1.74	1.75	1.46	1.31	1.66	1.54	0.002	0.10
	*Bacteroidetes*					21.7	19.2	18.4	18.9	20.7	20.9	19.01	0.019	0.15
		*Bacteroidia*				15.5	14.9	13.6	15.2	17.5	17.7	15.69	0.014	0.09
			*Bacteroidales*			15.5	14.9	13.6	15.2	17.5	17.7	15.69	0.014	0.09
				*Prevotellaceae*		6.13^b^	7.14^b^	6.57^b^	9.30^a^^b^	12.6^a^	12.3^a^	9.85^a^^b^	0.008	< 0.001
					*Prevotella*	4.26^c^	5.13^c^	4.66^c^	6.53^c^	9.55^a^	9.42^a^^b^	6.77^b^^c^	0.007	< 0.001
					*Paraprevotella*	0.41	0.47	0.42	0.75	0.38	0.56	0.45	0.001	0.21
					Unclassified*_Prevotellaceae*	1.40^b^	1.48^b^	1.43^b^	1.93^a^^b^	2.53^a^	2.26^a^^b^	2.53^a^	0.003	< 0.01
				*Porphyromonadaceae*		6.69^a^	5.10^a^^b^	4.49^a^^b^	3.53^b^	2.69^b^	3.50^b^	4.06^b^	0.008	< 0.01
				Unclassified*_Bacteroidales*		2.66^a^	2.74^a^^b^	2.58^a^^b^	2.35^a^^b^^c^	2.23^a^^b^^c^	1.84^b^^c^	1.77^b^	0.004	< 0.01
		Unclassified*_Bacteroidetes*				6.05	4.09	4.54	3.64	3.13	3.07	3.20	0.007	0.19
	*Proteobacteria*					6.87^b^	13.6^b^	24.1^a^^b^	20.6^a^^b^	32.8^a^	28.3^a^^b^	26.4^a^^b^	0.056	< 0.01
		*Gammaproteobacteria*				4.52^b^	11.7^a^^b^	22.0^a^^b^	19.1^a^^b^	31.6^a^	26.7^a^^b^	24.7^a^^b^	0.057	< 0.01
			*Aeromonadales*			3.06	6.45	5.05	4.66	4.25	3.79	4.25	0.013	0.19
				*Succinivibrionaceae*		3.06	6.45	5.05	4.66	4.25	3.79	4.25	0.014	0.19
					*Ruminobacter*	1.98	4.57	3.69	3.37	3.39	2.51	2.84	0.012	0.29
					*Succinimonas*	0.66^a^^b^	0.88^a^	0.82^a^^b^	0.54^a^^b^	0.37^a^^b^	0.34^b^	0.52^a^^b^	0.001	0.02
					*Succinivibrio*	0.41	0.97	0.51	0.73	0.47	0.92	0.88	0.002	0.12
			Unclassified*_Gammaproteobacteria*			1.39^b^	5.21^b^	16.8^a^^b^	14.4^a^^b^	27.3^a^	22.9^a^^b^	20.4^a^^b^	0.065	0.01
		*Alphaproteobacteria*				0.68	0.48	0.61	0.44	0.26	0.45	0.34	0.002	0.27
			Unclassified*_Alphaproteobacteria*			0.62	0.44	0.57	0.31	0.23	0.39	0.31	0.001	0.27
		Unclassified*__Proteobacteria*				1.26^a^	1.03^a^^b^	1.13^a^^b^	0.80^b^	0.68^b^	0.82^a^^b^	1.01^a^^b^	0.001	0.01
	*Fibrobacteres*					2.06	1.71	1.25	1.81	1.44	1.82	1.35	0.003	0.24
					*Fibrobacter*	2.06	1.71	1.25	1.81	1.44	1.82	1.35	0.003	0.24
	*Spirochaetes*					1.57	1.54	1.36	2.54	1.49	2.37	2.51	0.003	0.01
					*Treponema*	1.48	1.43	1.28	2.45	1.45	2.31	2.41	0.003	0.01
	Unclassified*_Bacteria*					6.75	5.70	5.77	5.06	4.53	4.79	4.68	0.006	0.06

^1^Number of sequences allocated to the individual taxa relative to the total number of sequences. For those sequences classified all the way from phylum to genus level, intermediate taxonomic identifications were omitted. Those bacterial phyla with relative abundance lower than 1% were not shown.

^2^Sample numbers for each time period were: W-3(10),W-2(9),W-1(10),W1(10),W2(9),W3(9),W4(13).

^3^Different taxonomic levels were indicated by the first letter of each (K: Kingdom; P:Phylum; C:Class; O:Order; F:Family; G:Genus).

^4^*P*-value smaller than 0.05 indicates the significant effect of fixed factor 'weeks relative to parturition'.

^a-c^Least squares means within a row with different superscripts differ significantly (*P* < 0.05); Mean standard error is presented

The *Bacteroidetes* phylum represented by the *Bacteroidia* class and the *Bacteroidales* order showed no significant changes over the transition period; nevertheless, two large families of this phylum, *Prevotellaceae* and *Porphyromonadaceae*, showed different patterns where the former increased significantly from ~6% to ~12% and the latter decreased significantly from ~7% to ~3% over the transition period. *Prevotella* (4.26%-9.55%) and *Paraprevotella* (0.38%-0.75%) were the predominant genera of the *Prevotellaceae* family; The *Prevotella* genus showed a significant increase in relative abundance from 4.26% to 9.55% over the transition period, whereas the *Paraprevotella* genus showed no significant change. Although with an overall low relative abundance, unclassified *Prevotellaceae* increased significantly from 1.4% in the prepartum period to 2.53% in the postpartum period.

The *Proteobacteria* phylum, dominated by the *Gammaproteobacteria* class, showed a significant increase from the lowest relative abundance of ~7% in the prepartum period to the highest relative abundance of ~33% in the postpartum period. The most abundant family of the *Aeromonadales* order (3.06%-6.45%), *Succinivibrionaceae*, showed no significant changes over the transition period, as did the two dominant genera, *Ruminobacter* (1.98%-4.57%) and *Succinivibrio* (0.41%-0.97%). With a low relative abundance (less than 1%), *Succinimonas* showed a significant decrease from the highest relative abundance of 0.88% in the prepartum period to the lowest relative abundance of 0.34% in the postpartum period. Despite changes in the classified groups, a significant increase in relative abundance was observed for the unclassified *Gammaproteobacteria* (1.39%-27.32%) and a significant decrease from 1.26% to 0.68% over the transition period was observed for the unclassified *Proteobacteria*.

The least abundant bacteria phyla, *Fibrobacteres* and *Spirochaetes*, including lower taxonomic groups within these phyla, showed no significant changes over the transition period.

### Diversity analysis of the active bacterial community

**Fig 3:**
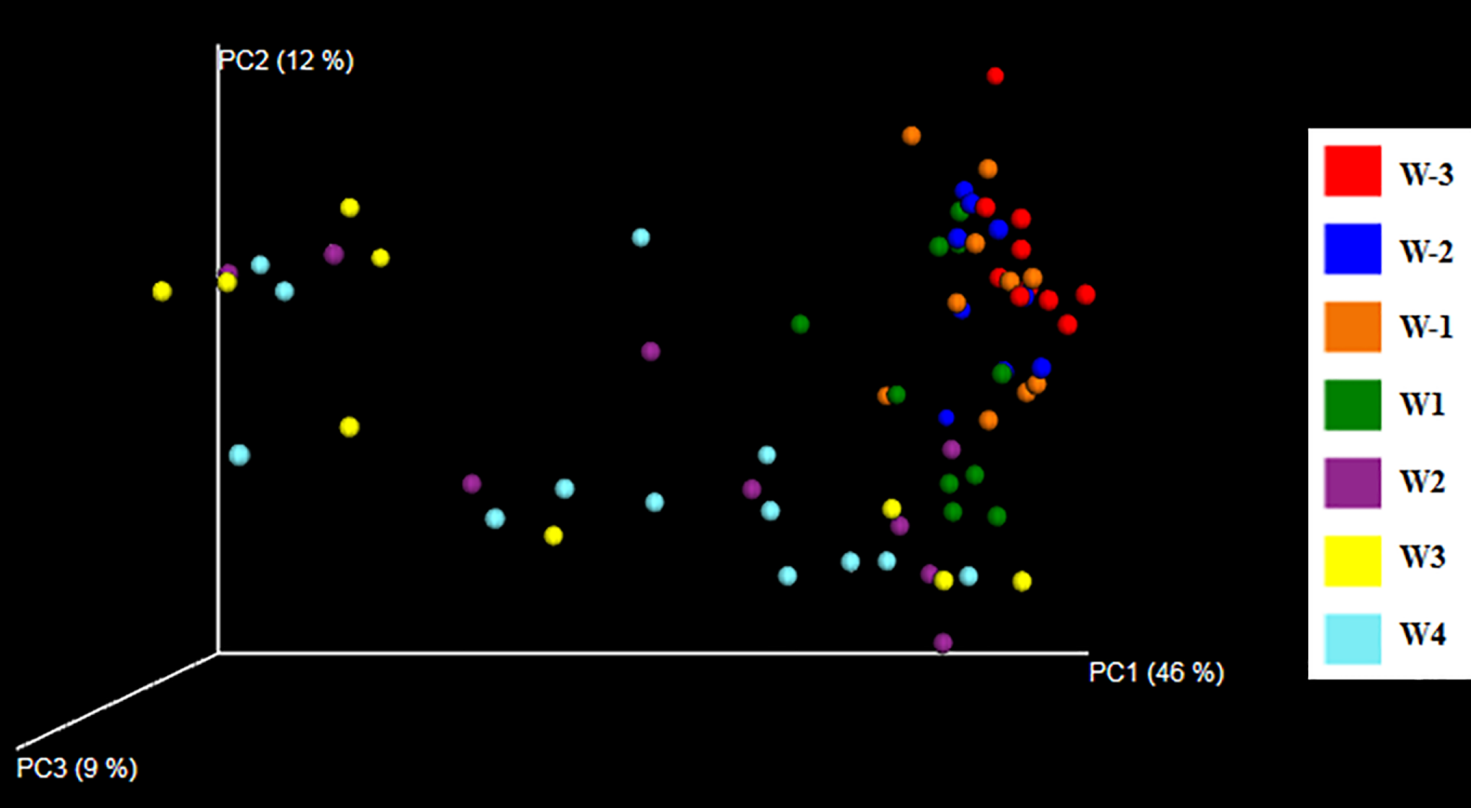
Principal coordinate analysis (PCoA) of the active rumen bacterial community. The weighted UniFrac distance matrix was used for the analysis of the bacterial 16S rRNA transcript amplicons. Weekly based sample groups are indicated by different colors.

The species richness (Chao1) of the bacterial community decreased significantly over the transition period with the highest value (3708.15) in the three weeks before parturition (W-3) and the lowest value (3045.70) in the third week after parturition (W3) (Table 2). The number of observed species was significantly reduced in the last two weeks after parturition (W3 and W4) compared with the three weeks before parturition (W-3). Similar decreases from the three weeks before parturition (W-3) to the second and third week after parturition (W2 and W3) were observed for the phylogenetic diversity (PD_whole_tree) of the active bacterial community. Principal coordinate analysis (PCoA) showed dynamic shifts of the bacterial community over the transition period, as seen when plotting the first two principal coordinates explaining 58% of the variation (Fig 3). The entire bacterial community showed significant shifts over the transition period as evaluated by weighted UniFrac distance matrix based ANOSIM test (R = 0.29, *P*-value = 0.001).

**Table 2:** Alpha diversity analysis of the rumen active methanogen and bacterial communities.

Item^2^	Weeks relative to parturition^1^							SEM	*P*-value^3^
	W-3	W-2	W-1	W1	W2	W3	W4		
***mcrA***									
Chao1	59.2	58.3	58.2	58.5	52.7	55.6	59.5	2.16	0.150
Observed_species	54.3	52.8	53.5	51.9	51.6	51.9	52.2	1.76	0.792
PD_whole_tree	2.19	2.21	2.25	2.17	2.19	2.19	2.20	0.08	0.709
**Bacterial 16S rRNA**									
Chao1	3708^a^	3495^a^^b^	3424^a^^b^^c^	3340^a^^b^^c^	3135^b^^c^	3045^c^	3386^a^^b^^c^	129.71	0.002
Observed_species	2993^a^	2695^a^^b^	2669^a^^b^	2553^a^^b^	2476^a^^b^	2372^b^	2700^b^	129.00	0.012
PD_whole_tree	171.3^a^	156.8^a^^b^	155.4^a^^b^	149.8^a^^b^	145.4^b^	140.1^b^	154.6^a^^b^	5.85	0.005

^1^Weekly based samples groups are indicated and sample numbers for each time period were: W-3(10), W-2(9), W-1(10), W1(10), W2(9), W3(9), W4(13).

^2^ Alpha diversity (Chao1, Observed species and PD whole tree) analysis was performed for the active methanogen community profiled by Illumina amplicon sequencing of *mcrA* transcripts, and the active bacterial community profiled by Illumina amplicon sequencing of bacterial 16S rRNA transcripts.

^3^*P*-value smaller than 0.05 indicates the significant effect of fixed factor ‘Weeks relative to parturition’.

^a-c^Least square means (t-test) within a row with different superscripts differ significantly (*P* < 0.05); Mean standard error is indicated.

### Archaeal 16S rRNA transcript amplicon profile

With the universal prokaryotic 16S rRNA gene primer targeting both the bacterial and archaeal communities, nearly 3% of the total sequences were assigned to the *Euryarchaeota* phylum of the *Archaea* domain (Fig 2). The *Euryarchaeota* phylum was composed of two major orders, *Methanobacteriales* (80.6%-89.7%) and *Methanomassiliicoccales* (2.42%-14.8%), showing no significant changes over the transition period (S2 Fig and Table 3). Within the *Methanobacteriales* order, two predominant genera of the ruminal active archaeal community were *Methanobrevibacter* (61.8%-69.8%) and *Methanosphaera* (8.16%-18.5%). Sequences assigned to the *Methanomassiliicoccales* order (2.42%-14.8%) were further allocated to an unclassified group. The *Methanobacteriales* order accounting for 80%-90% of the active archaeal community was more abundant compared with the *Methanomassiliicoccales* order which varied a lot in relative abundance ranging from 2% in the postpartum period to 15% in the prepartum period. The 16S rRNA transcript profile of all archaeal taxa showed significant changes over the transition period; the *Methanobacteriales* order and the *Methanosphaera* genus increased significantly in relative abundance from ~80% to ~90%, and ~8% to ~18%, whereas the *Methanobrevibacter* genus (from 69.8% to 61.8%) and the *Methanomassiliicoccales* order (from 14.8% to 2.42%) decreased significantly over the transition period, as reflected by their highest and lowest values (Table 3).

**Table 3:** Community structure of the active methanogen community as revealed by Illumina amplicon sequencing of *mcrA* and bacterial 16S rRNA transcripts.

Item^2^	Weeks relative to parturition^1^							SEM	*P*-value^3^
	W-3	W-2	W-1	W1	W2	W3	W4		
***mcrA***									
*Methanobacteriales*	98.9	98.8	98.9	98.8	95.6	98.9	98.8	0.01	0.301
*Methanobrevibacter*	86.3^a^	81.3^a^	79.5^a^	69.8^b^	66.4^b^	70.4^b^	67.1^b^	2.10	< 0.001
*Methanosphaera*	12.6^b^	17.6^b^	19.4^b^	29.0^a^	29.2^a^	28.6^a^	31.7^a^	2.20	< 0.001
*Methanomassiliicoccales*	0.01	0.01	0.01	0.03	0.03	0.05	0.07	0.00	-
**Archaeal 16S rRNA**									
*Methanobacteriales*	80.6^b^	82.8^a^^b^	83.8^a^^b^	84.7^a^^b^	88.3^a^	89.7^a^	89.2^a^^b^	0.02	0.033
*Methanobrevibacter*	67.4^a^^b^	69.8^a^	67.6^a^^b^	61.8^b^	66.5^a^^b^	68.3^a^^b^	68.1^b^	0.02	0.017
*Methanosphaera*	9.96^b^^c^	8.16^c^	10.9^b^^c^	18.4^a^	18.5^a^	17.8^a^	17.5^a^^b^	0.02	< 0.001
*Methanomassiliicoccales*	14.8^a^	11.6^a^	10.3^a^^b^	6.81^b^^c^	4.92^c^	2.42^c^	2.47^c^	0.01	< 0.001

^1^ Weekly based samples groups are indicated and relative abundance (%) of individual methanogen group at the order and genus levels are displayed.

^2^ The proportion of unassigned reads is not shown in the *mcrA* profile due to its low number. It should be noted that archaeal 16S rRNA transcripts accounted for approx. 3% of the total 16S rRNA transcripts and were treated as 100% below.

^3^*P*-value smaller than 0.05 indicates the significant effect of fixed factor ‘Weeks relative to parturition’.

^a-c^Least squares means within a row with different superscripts differ significantly (*P* < 0.05); Mean standard error is presented.

### Quantifications of total methanogen and two major methanogen orders by qPCR

**Fig 4:**
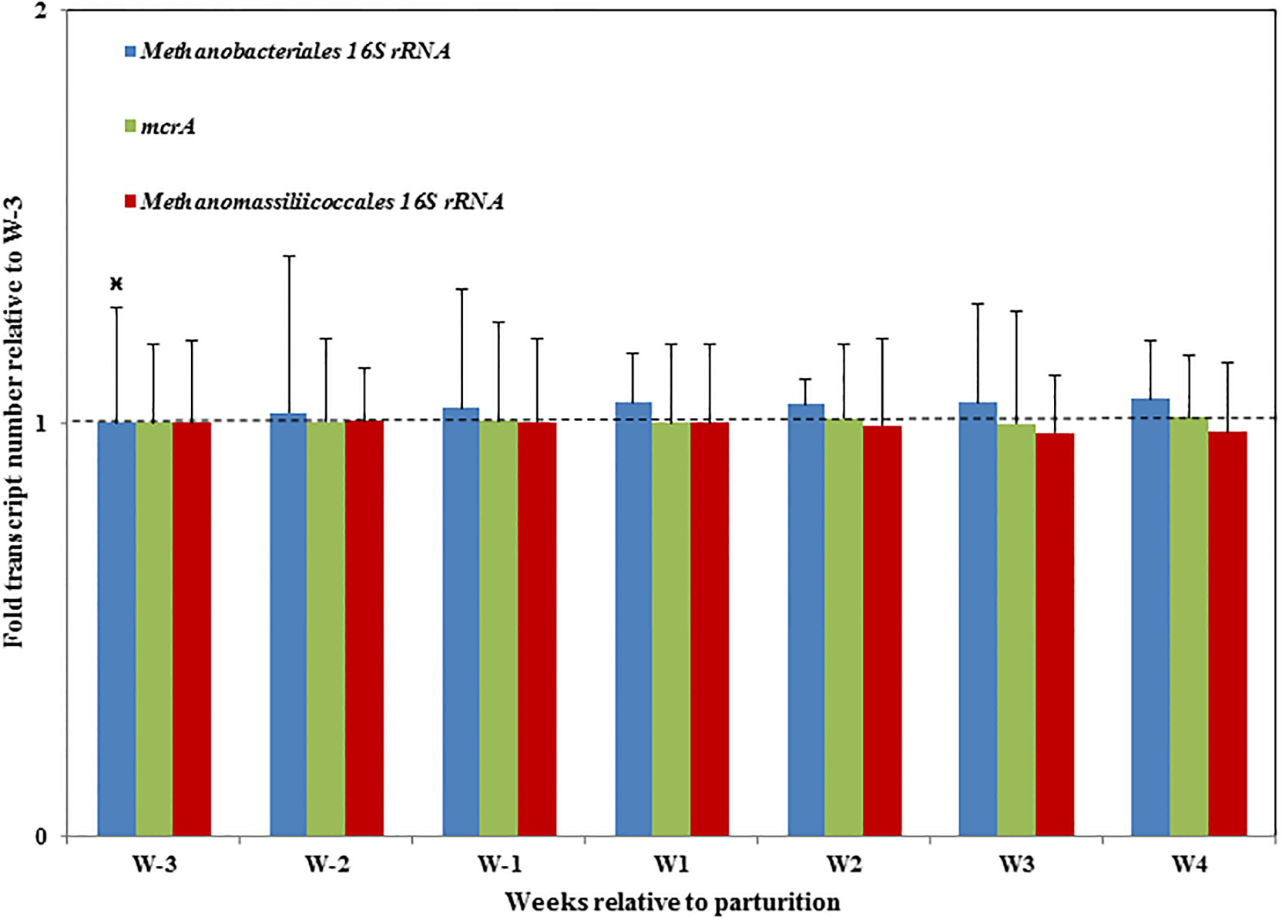
Quantification of total methanogen and two major methanogen orders by qPCR. Total *mcrA* transcript number, 16S rRNA transcript number of the *Methanomassiliicoccales* order and 16S rRNA transcript number of the *Methanobacteriales* order are quantified by qPCR. Fold change in transcript copy number was calculated relative to the third week before parturition (W-3). For *Methanobacteriales* 16S rRNA transcript number, the first group (W-3) was significant lower than the remaining six groups (*P* < 0.05), as marked by the asterisk.

The total *mcrA* transcript number showed no significant changes over the transition period (Fig 4). The 16S rRNA transcript number of *Methanomassiliicoccales* showed a decreasing trend over the transition period (Fig 4). Furthermore, *Methanobacteriales* showed a significantly lower 16S rRNA copy number of in the third week before parturition (W-3) in comparison with the remaining six weeks (Fig 4).

### T-RFLP analysis of *mcrA* transcripts

Seven dominant T-RFs were detected across all seventy samples, accounting for more than 89% of the total peak height. Among these dominant T-RFs (39, 105, 154, 267, 297, 385 and 475 bp), T-RFs 39 and 267 bp were the most abundant two fragments with a similar mean relative abundance of 38%, followed by T-RFs 475 (5%), 105 (3.6%) and 385 bp (3.3%); T-RFs 154 and 297 bp were the least abundant fragments (less than 1%) (Table 4). Across the entire study period, the most abundant five T-RFs showed no significant changes, whereas the least abundant two T-RFs, T-RF 154 and 297 bp, significantly decreased in relative abundance. Compared with the same analysis of *mcrA* gene (DNA) which produced 12 T-RFs (manuscript in preparation), *mcrA* transcripts (cDNA) gave us a smaller number of T-RFs (only seven here). Moreover, T-RFs 202 and 210 bp were present in DNA detection while absent from cDNA detection. Principal component analysis (PCA) of T-RFs profile showed no clear shifts of the active methanogen community over the transition period (S3 Fig).

**Table 4:** Relative abundance^1^ (%) of predominant terminal-restriction fragments (T-RFs) from *mcrA* transcript T-RFLP profiles across the transition period.

T-RFs (bp)	Weeks relative to parturition^2^							SEM	*P*-value^3^
	W-3	W-2	W-1	W1	W2	W3	W4		
39	37.3	38.0	36.7	39.5	38.1	38.2	39.2	1.10	0.359
105	3.71	3.44	3.39	3.40	3.86	3.95	3.24	0.45	0.844
154	0.9^a^	0.91^a^	0.87^a^	0.55^a^^b^	0.51^a^^b^	0.43^b^	0.41^b^	0.11	0.000
267	37.4	37.9	36.7	39.3	37.9	37.8	38.8	1.09	0.475
297	1.47^a^	1.35^a^	1.04^a^^b^	0.57^b^^c^	0.43^c^	0.49^b^^c^	0.21^c^	0.16	< 0.001
385	3.93	4.08	3.21	2.59	2.79	3.09	1.78	0.47	0.003
475	5.01	4.51	4.77	4.11	5.66	5.99	3.90	0.69	0.061

^1^Relative abundance is calculated as individual peak (T-RF) height relative to total peak height in each sample.

^2^Weekly based samples groups are indicated and sample numbers for each time period were: W-3(10), W-2(9), W-1(10), W1(10), W2(9), W3(9), W4(13).

^3^*P* value smaller than 0.05 indicates the significant effect of fixed factor ‘Weeks relative to parturition’.

^a-c^Least squares means within a row with different superscripts differ significantly (P < 0.05); Mean standard error is presented.

### *mcrA* transcript amplicon profile

A total of 85 OTUs were generated from the *mcrA* transcript profile after filtering out OTUs with sequence numbers lower than 3. The OTUs were taxonomically associated with two major orders of the active methanogen community, of which *Methanobacteriales* accounted for nearly 99% of the total sequences and *Methanomassiliicoccales* was relatively low (less than 0.1% of total sequences). The most abundant genera observed were *Methanobrevibacter* (75%), *Methanosphaera* (24%) and a *Methanomassiliicoccales*-related genus (less than 1%); their respective mean relative abundance are shown in brackets (Table 3). On the other hand, there were nearly 1% of the total sequences unassigned. Although the *Methanobacteriales* order showed no significant change over the transition period, the *Methanobrevibacter* genus of this order showed a significant decrease from the highest relative abundance of 86.3% in the prepartum period to the lowest abundance of 66.4% in the postpartum period. The *Methanosphaera* genus on the other hand, showed a significant increase from the lowest relative abundance of 12.6% in the prepartum period to the highest relative abundance of 31.7% in the postpartum period.

### Diversity analysis of the active methanogen community

**Fig 5:**
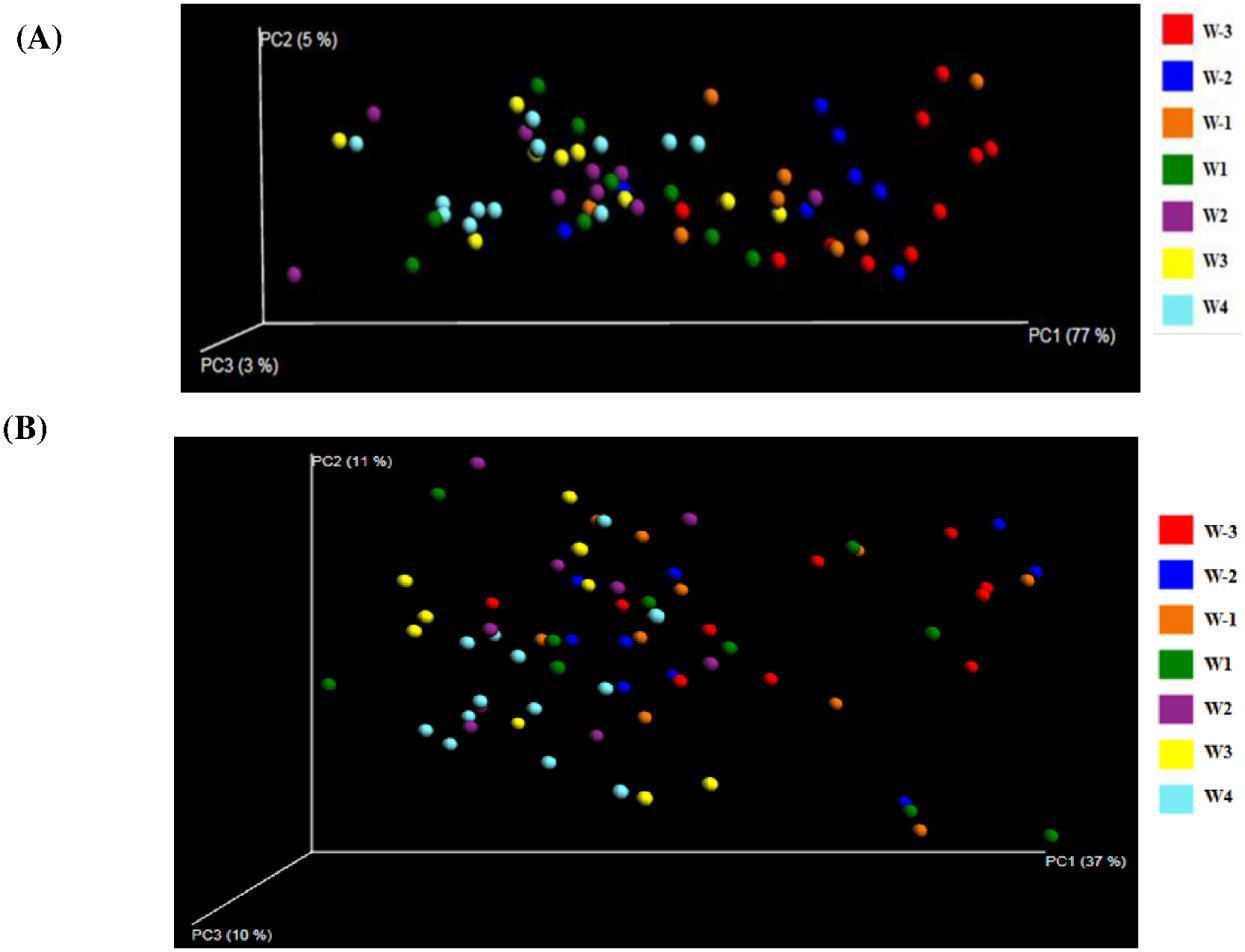
Principal coordinate analysis (PCoA) of the active rumen archaeal (methanogen) community. Weighted UniFrac distance matrix was used for the PCoA analysis of the archaeal (methanogen) community, and *mcrA* transcript amplicon profile (A) and archaeal 16S rRNA transcript amplicon profile (B) based analysis were presented, respectively. Weekly based sample groups are indicated by different colors.

No significant change was observed for the three indicators of alpha diversity (chao1, observed species and PD_whole_tree) according to *mcrA* transcript amplicon profile (Table 2). However, weighted UniFrac distance matrix based principal coordinate analysis (PCoA) indicated a clear shift of the active methanogen community along the first principal coordinate (77%), as demonstrated by ANOSIM test (R = 0.28, *P* = 0.001) (Fig 5).

### Phylogeny of the active methanogen community

**Fig 6:**
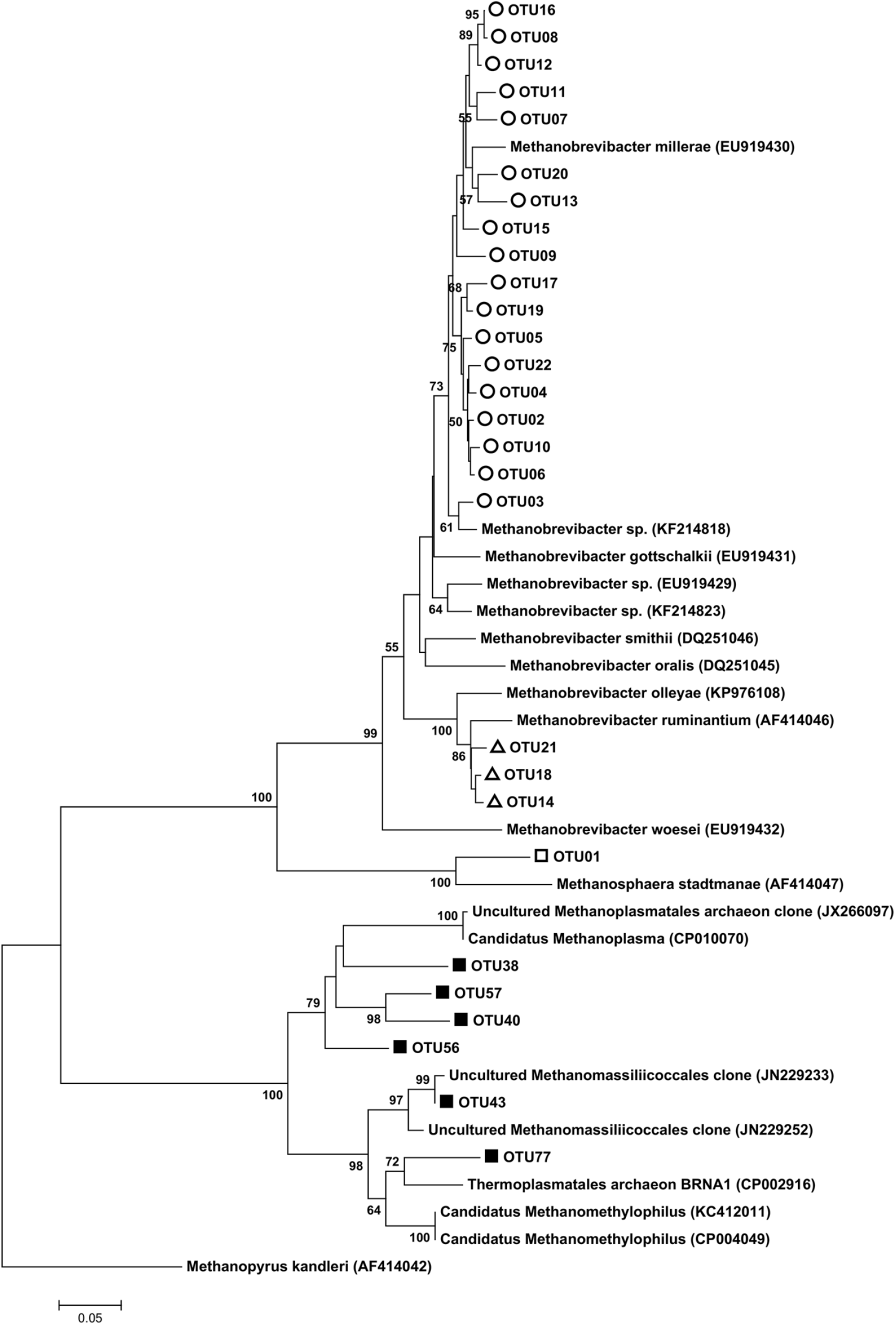
Phylogeny of the *Methanobacteriales*-related OTUs and *Methanomassiliicoccales*-related OTUs. Representative *mcrA* sequences from the most abundant *Methanobacteriales*-related OTUs (22 OTUs) and *Methanomassiliicoccales*-related OTUs (6 OTUs) were picked out for the construction of the phylogenetic tree, and reference *mcrA* gene sequences were downloaded from the NCBI nucleotide database. The tree was created using the Neighbor-joining method with 500 re-assemblies; bootstrap values greater than 50 are shown. Three *Methanobacteriales* clades are indicated by different symbols: the *Methanosphaera* clade (squares), the *Methanobrevibacter ruminantium* clade (triangles) and the *Methanobrevibacter gottschalkii* clade (circles). The *Methanomassiliicoccales* clade is indicated by filled squared.

The most abundant 22 OTUs related to the *Methanobacteriales* order were clustered into three clades on the phylogenetic tree, namely *Methanosphaera* clade, *Methanobrevibacter ruminantium* clade and *Methanobrevibacter gottschalkii* clade (Fig 6). Six *Methanomassiliicoccales* related OTUs were clustered into one clade, however the gastrointestinal tract (GIT) clade and environmental clade were proposed in a recent report regarding the phylogeny of *Methanomassiliicoccales* [36] (Fig 6).

## Discussion

### The rumen active bacterial community

In line with the statement of ‘core microbiomes’ in previous studies[5,37–39], we here propose a core microbiome of rumen active bacteria composed of 22 genera ubiquitously present in the 16S rRNA dataset of all rumen samples, with *Ruminococcus*, *Prevotella*, *Ruminobacter*, *Fibrobacter* and *Butyrivibrio* being the most dominant. Overall, *Firmicutes* (~46% of total reads) was the most abundant phylum followed by *Bacteroidetes* and *Proteobacteria* with similar abundances (~20%). By comparative microarray and qPCR analysis of DNA and RNA derived materials, *Proteobacteria* was reported as the most active group of the rumen bacterial community in beef steers[9], and amplicon sequencing of bacterial 16S rRNA revealed *Proteobacteria* as the predominant phylum, accounting for approx. 46% of the rumen bacterial community in beef steers[13]. The discrepancy between the present and the previous studies regarding the *Proteobacteria* proportion might be due to differences in diets and animal breeds; grain-based diet were fed to adult Brahman-cross steers [9] and high-energy finishing diet were fed to ten-month crossbred beef steers[13]. In the present study, a significant increase in the proportion of *Proteobacteria* was observed from the prepartum (6–8%) to the postpartum (32–33%) period in the 16S rRNA dataset, suggesting that *Proteobacteria* could be favored and more active in the rumen bacterial community of animals fed high-concentrate diets. Additionally, we observed decreasing trends from the prepartum to the postpartum period for unclassified *Bacteroidales* and *Ruminococcaceae*, suggesting that unclassified *Bacteroidales* and *Ruminococcaceae* are more active in animals fed the prepartum diet. This is in accordance with a DNA-based study, where unclassified *Bacteroidales* and *Ruminococcaceae* were reported to be abundant groups in animals fed forage-based diets[37].

### Diversity of the rumen active bacterial community

Over the transition period, we observed a decreased bacterial diversity at the RNA as well as the DNA level. It has been reported that high concentrate diets support low bacterial diversity and, vice versa, high forage diets support high bacterial diversity in the rumen[40,41]. Therefore, dietary shifts over the transition period are likely to be responsible for the decreased bacterial diversity observed here. In line with our observations, a DNA based study showed a decrease in the rumen bacterial diversity (Chao1 and Shannon diversity indexes) from the prepartum to the postpartum period[5]. This could imply that host physiological stage may be also involved in the alteration of bacterial diversity. Collectively, we revealed the shifts of the rumen bacterial community over the transition period in both the DNA and RNA based approach, as illustrated by the beta-diversity analysis (PCoA plot),

### The rumen active archaeal community

*Euryarchaeota* was the most dominant archaeal phylum in the prokaryotic 16S rRNA profile. Further, in accordance with DNA based approaches[14,15,42], the RNA based approach in the present study revealed *Methanobacteriales* and *Methanomassiliicoccales* as the two major archaeal orders and *Methanobrevibacter* and *Methanosphaera*, both *Methanobacteriales*, as the major genera. The dominance of these two orders was further verified by qPCR (16S rRNA transcript numbers). The RNA amplicon sequencing of prokaryotic 16S rRNA and *mcrA* revealed similar archaeal community structure as the DNA based amplicon sequencing (manuscript in preparation); a finding that was further supported by the T-RFLP profiles generated from *mcrA* gene sequences (DNA) and *mcrA* transcripts (RNA), indicating accordance between presence and activity of the archaeal community members.

Phylogenetic analysis of *mcrA* sequences revealed three clades of the order *Methanobacteriales*, *Methanobrevibacter gottschalkii* clade, *Methanobrevibacter ruminantium* clade and *Methanosphaera*, contributing more than 95% to the rumen active methanogen communities. This observation is comparable with results from DNA (16S rRNA gene) based analysis[15]. The order *Methanobacteriales* consisted of *Methanobrevibacter* and *Methanosphaera* (< 5%) in a DNA based study, accounting for > 98% of the rumen archaeal communities in dairy cows[43]. The dominance of *Methanobacteriales* has further been reported in several earlier studies [15,42,44].

According to the RNA amplicon sequencing (prokaryotic 16S rRNA) results, the *Methanomassiliicoccales* accounted for 2.4%-15% of the total archaeal 16S rRNA, similar to the DNA level proportion (10.4%) reported in New Zealand sheep and cattle[15]. However, the *Methanomassiliicoccales* comprised a very low proportion (< 1%) of the *mcrA* transcript amplicons as well as the T-RFLP profile of *mcrA* transcripts, indicating the *Methanomassiliicoccales* order to be low in abundance. This shows that there is a discrepancy between the proportion of *Methanomassiliicoccales*-related 16S rRNA and *mcrA*. The *mcrA* gene, encoding the alpha-subunit of the methyl-coenzyme M reductase, has been chosen as a marker for methanogens [45] and has been widely used in clone library based analyses to characterize the community structure of methanogens[46,47]. However, the use of the *mcrA* gene for taxonomy and phylogeny based work is questionable mainly because a comprehensive *mcrA* gene reference database is still not available. Thus, *Methanomassiliicoccales* was detected in the methanogen communities of anaerobic digester as evaluated by the archaeal 16S rRNA gene profile, but was not detected when the *mcrA* gene was used as a phylogenetic marker[48]. Therefore, it still seems critical to use the *mcrA* gene as a phylogenetic marker for the classification of the *Methanomassiliicoccales* group. Although less representative in the *mcrA* sequencing profile, *Methanomassiliicoccales*-related OTUs can be classified into two clades in the present study, namely the gastrointestinal tract (GIT) and the environmental clade, as previously suggested[36]. The poor detection of this group seems unlikely to be caused by the sequencing depth, since the Illumina MiSeq platform rendered higher *mcrA* read numbers (approx. 16,000) than 454 pyrosequencing platform (approx. 5,000) [48]. The detection of *Methanomassiliicoccales* related species in the rumen were largely influenced by different sequencing and analysis methods and the *Methanomassiliicoccales* group was undetected by Illumina amplicon sequencing of archaeal 16S rRNA gene and Illumina metagenomics sequencing of *mcrA*, as illustrated by Snelling et al. (2014)[14]. Overall, isolation and characterization of *Methanomassiliicoccales* members are thus highly required for further investigating and defining their role in the rumen ecosystem.

### Associative patterns of the rumen active methanogen community over the transition period

The observation of a constant level (qPCR) of total methanogens across the transition period is in line with an investigation across animal species and diets [42] as well as comparison between high and low methane emitters[17]. Compared with the T-RFLP profile, more shifts were revealed by the RNA amplicon sequencing of prokaryotic16S rRNA and *mcrA*, due to the massive amount of information provided by the next generation sequencing technology.

The *Methanobrevibacter* genus, dominated by typical formate-, H_2_- and CO_2_-utilizing hydrogenotrophs, decreased in relative abundance of the active Archaea across the transition period. *Methanobrevibacter* phylotypes have been reported to be predominantly present in the rumen of cattle fed on high fibrous diets containing wheat straw[46]. The production of H_2_ is strongly associated with the degradation of fibrous plant material, therefore affecting the activity of methanogens[49]. Thus, in our case, the low forage content in the postpartum diet might be responsible for the decrease in relative abundance of *Methanobrevibacter*. *Methanosphaera*, on the other hand, was more abundant in the postpartum period. Representative *Methanosphaera* members, like *Methanosphaera stadtmanae*, are hydrogen-dependent methylotrophs, producing methane by reducing methanol with hydrogen as electron donor. The opposite trends observed for the *Methanobrevibacter* and *Methanosphaera* genera suggest that there might be competition for H_2_ between these two groups. Additionally, it has been reported that *Methanosphaera stadtmanae* was significantly more abundant in the rumen of beef cattle fed low forage diet[50]. The shift from a high-forage prepartum diet to a low-forage postpartum diet might explain the observed increase in *Methanosphaera*. Besides, the postpartum diet contained 11.19% sugar beet pellets which could be an abundant source of pectin for the production of methanol, supporting the growth of *Methanosphaera* species. The significant decrease of the *Methanomassiliicoccales* group was likely due to the dietary supplementation of 11.19% of 10.5% rapeseed fat in the postpartum diet, as supported by a previous study[18].

### Diversity of the rumen active methanogen community

The same dominant archaea were present in the rumen methanogen communities of farmed sheep, cattle and red deer fed different diets[42]. There are several studies indicating that the methanogen communities are resistant to dietary changes when shifting e.g. from a high forage to a high grain diet[43,51]. This might explain our observation that there was no significant change in the alpha diversity indexes (chao1, observed species and phylogenetic diversity) in the RNA amplicon sequencing profile of *mcrA*. However, the observed shifts in beta diversity analysis as illustrated by PCoA plot and the dynamic shifts in the community composition of methanogen communities might be due to the low abundance OTUs. It has been suggested that there are diet- and ruminant- species-based differences in the archaeal community structure[42]. The differences in methanogen community composition could result from differences in animal species, animal age, diet type, and environment[52]. By using 16S rRNA gene clone library methods, Wright et al. (2004) found the effect of diet on the diversity of the methanogen community. The diversity of the methanogen communities was higher in multiparous cows (higher Shannon index value) than in primiparous cows[43], implying that age is also a factor influencing the diversity of rumen methanogen community.

After calving, dairy cows appear to be in negative energy balance as indicated by a decrease in blood glucose followed by an increase of non-esterified fatty acid and β-hydroxybutyrate[53]. In accordance with this, we observed a dramatic increase in dry matter intake of dairy cows after calving as well as a linear increase in ruminal capacity and dry matter fill has been observed during the early lactation period[54]. These host physiology associated factors could shape the rumen microbial community e.g. by affecting fast and slow growing microbes distinctly.

We observed significant shifts of the rumen microbial community over seven consecutive weeks during the transition period, with a relative low number of animals compared to other studies[3–5]. Recognizing that the power of a study like the present could be increased by including more animals, we are however always forced to take the principle of the three Rs’ (Replace, Reduce, Refine) into consideration. Moreover, to exclude potential biases related to the flora scoop rumen sampling procedure, use of rumen fistulated animals could be considered.

## Conclusion

The rumen metabolically active bacterial community of dairy cows were analyzed over the transition period by RNA amplicon sequencing (Illumina MiSeq) of prokaryotic 16S rRNA. *Firmicutes* (35%-57%) was the most abundant phylum of the rumen active bacterial community. The most frequently detected genera, *Ruminococcus*, *Ruminobacter* and *Fibrobacter*, were not only dominating active members of the rumen bacterial community but were also more or less unaffected by the dietary shifts over the transition period. In response to the transition period, members of the rumen active bacterial community of dairy cows showed different patterns. RNA amplicon sequencing of 16S rRNA and *mcrA* was applied for characterizing the metabolically active rumen archaeal community, which was dominated by two major orders, *Methanobacteriales* (mainly *Methanobrevibacter* and *Methanosphaera*) and *Methanomassiliicoccales*, showing significant shifts over the transition period. However, the use of the *mcrA* gene for taxonomy studies must be considered carefully since primer biases and database limitations might lead to poor detection of e.g. the *Methanomassiliicoccales* members.

## Supporting information

S1 Fig Dry matter intake (DMI) over the transition period. Dry matter intake (kg/day) was recorded daily for each cow. Data are presented as average value (+/- SE) of each week for all cows pooled into weeks relative to parturition.
(JPG)

S2 Fig Rumen active archaeal community composition at order and genus levels. The archaeal 16S rRNA sequences comprised approx. 3% of the prokaryotic 16S rRNA amplicons. The archaeal community was made up of four orders *Nitrosphaerales*, *Thermoplasmatales*, *Methanosarcinales* and *Methanobacteriales* and the overall composition at the genus level is shown. The bars represent the weekly based sample groups.
(TIF)

S3 Fig Principal component analysis (PCA) of the relative abundance of predominant T-RFs. The relative abundance of predominant T-RFs identified in the T-RFLP profile was used for the analysis and weekly based sample groups indicated by different shapes either filled or unfilled are shown.
(TIF)

S1 Table Ingredients and chemical composition of the total mixed rations (TMR) fed prepartum and postpartum period. (DOCX)

S2 Table Primer pairs used for quantifying the absolute abundance of total methanogen and specific methanogen groups. (DOCX)
