# Supplementary figures and images for: Community structure of the metabolically active rumen bacterial and archaeal communities of dairy cows over the transition period
Source: PLoS One. 2017 Nov 8;12(11):e0187858. doi: 10.1371/journal.pone.0187858 (PMC5678694; doi:10.1371/journal.pone.0187858)

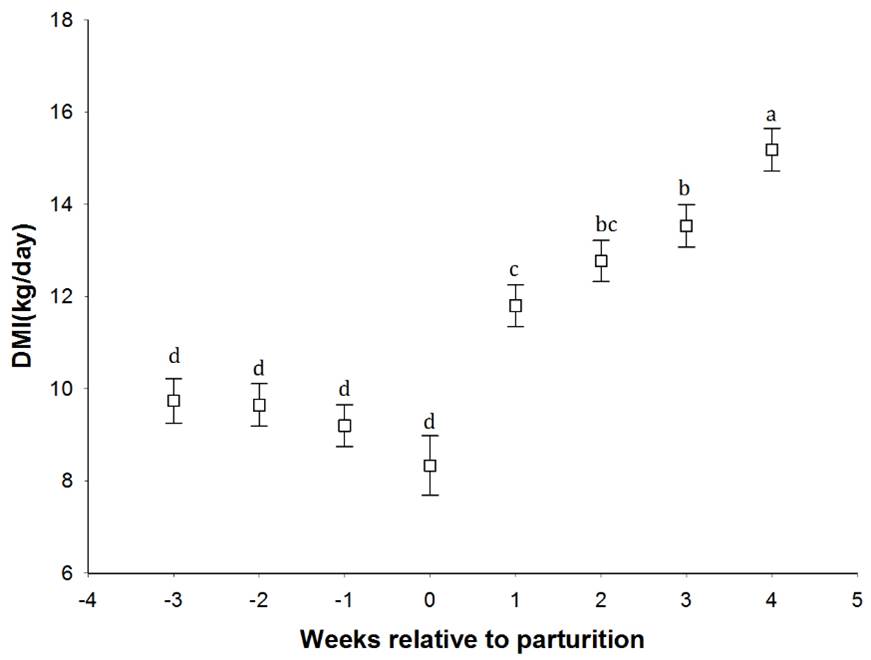

Supplement: S1 Fig — Dry matter intake (kg/day) was recorded daily for each cow. Data are presented as average value (+/- SE) of each week for all cows pooled into weeks relative to parturition. (JPG) [file pone.0187858.s001.jpg]

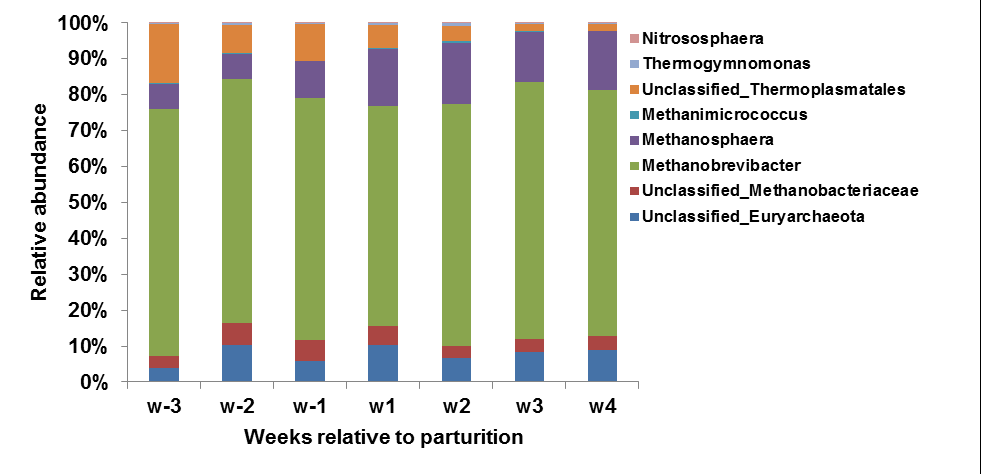

Supplement: S2 Fig — The archaeal 16S rRNA sequences comprised approx. 3% of the prokaryotic 16S rRNA amplicons. The archaeal community was made up of four orders Nitrosphaerales, Thermoplasmatales, Methanosarcinales and Methanobacteriales and the overall composition at the genus level is shown. The bars represent the weekly based sample groups. (TIF) [file pone.0187858.s002.tif]

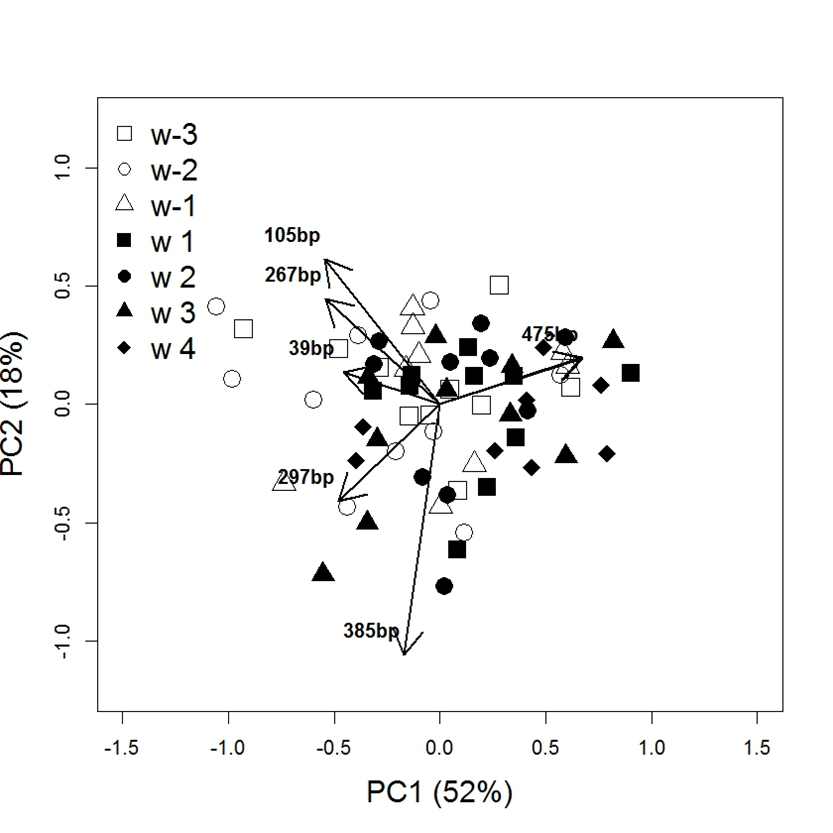

Supplement: S3 Fig — The relative abundance of predominant T-RFs identified in the T-RFLP profile was used for the analysis and weekly based sample groups indicated by different shapes either filled or unfilled are shown. (TIF) [file pone.0187858.s003.tif]
