# Supplementary material for: Community structure of the metabolically active rumen bacterial and archaeal communities of dairy cows over the transition period
Source: PLoS One. 2017 Nov 8;12(11):e0187858. doi: 10.1371/journal.pone.0187858 (PMC5678694; doi:10.1371/journal.pone.0187858)
Supplement: S1 Table — (DOCX) [file pone.0187858.s004.docx]

**S1 Table. Ingredients and chemical composition of the total mixed rations (TMR) fed prepartum and postpartum period.**

| Item, dry matter composition (%) | Prepartum | Postpartum |
| --- | --- | --- |
| Spring barley |  | 9.85 |
| NaOH treated wheat |  | 9.85 |
| Rapeseed meal I 4% fat | 8.47 |  |
| Rapeseed cake II 10.5% fat |  | 11.19 |
| Sugar beet pellets (un-molassified) |  | 11.19 |
| Grass silage I^1^ |  | 22.38 |
| Maize silage^2^ | 44.03 | 25.52 |
| Grass silage II^3^ | 25.40 | 8.95 |
| Spring barley straw | 21.17 |  |
| Urea |  | 0.16 |
| Feed salt |  | 0.18 |
| Cowmix (Komix) 302-318^4^ |  | 0.72 |
| Cowmix (Komix) gold^5^ | 0.93 |  |

^1^ Chemical analysis: DM, 29.93%; Ash, 10.16% of DM; Fiber, 22.02% of DM; NDF, 36.08% of DM; Soluble Nitrogen 1.79% of DM; Fat, 4.13% of DM; Total sugar, 0.33% of DM; Rumen soluble Organic Matter, 81.48% of DM; pH=4.

^2^Chemical analysis: DM, 24.59%; Ash, 3.28% of DM; Fiber, 25.08% of DM; NDF, 49.7% of DM; Soluble Nitrogen, 0.71% of DM; Fat, 2.13% of DM; Starch, 19.86% of DM; Rumen soluble organic matter, 70.72% of DM; pH=3.8.

^3^Chemical analysis: DM, 23.51%; Ash, 9.6% of DM; Fiber, 29.44% of DM; NDF, 48.99% of DM; Soluble Nitrogen, 1.27% of DM; Starch, 2.03% of DM; Fat, 2.9% of DM; Total sugar, 0.22% of DM; Rumen soluble organic matter, 71.79% of DM; pH=3.9.

^4^Cowmix 302-318 (Mineral) containing (100 gram): Ca, 30.3%; Mg, 7.8%; Sulfur, 1.3%.

^5^Cowmix gold (Mineral) containing (1 kilogram): Ca, 1.65%; Mg, 19.33%; P, 3.05%; K, 0.3%; Na, 3.7%.
