# Supplementary material for: Community structure of the metabolically active rumen bacterial and archaeal communities of dairy cows over the transition period
Source: PLoS One. 2017 Nov 8;12(11):e0187858. doi: 10.1371/journal.pone.0187858 (PMC5678694; doi:10.1371/journal.pone.0187858)
Supplement: S2 Table — (DOCX) [file pone.0187858.s005.docx]

| **S2 Table. Primer pairs used for quantifying the absolute abundance of total methanogen and specific methanogen groups.** | | | | | |
| --- | --- | --- | --- | --- | --- |
| Primer pairs | Targeting | Sequence (5’ → 3’) | Frag length (bp) | Annealing temperature | Reference |
| AS1-F | *Methanomassiliicoccales* | CAG CAG TCG CGA AAA CTT C | 485 (481) | 60 | [1] |
| AS2-R |  | AAC AAC TTC TCT CCG GCA C |  |  | And also used in [2] |
| mcrA_F | *Methanogens* | GGY GGT GTM GGD TTC ACM CAR TA | 469 | 60 | [3] |
| mcrA-R |  | CGT TCA TBG CGT AGT TVG GRT AGT |  |  |  |
| 915F | *Methanobacteriales* | AGG AAT TGG CGG GGG AGC AC | 195 | 60 | [4] |
| 1100AR |  | TGG GTC TCG CTC GTT G |  |  |  |
| 341F | *Prokaryotes* | CCT AYG GGR BGC ASCAG | 466 | 60 | [5] |
| 806R |  | GGA CTA CNN GGG TAT CTA AT |  |  | [6] |
| mcrA-F | *Methanogen* | TCGTCGGCAGCGTCAGATGTGTATAAGAGACAG-GGTGGT GTMGGDTTYACHCARTA | 475 | 60 | [3] |
| mcrA-R |  | GTCTCGTGGGCTCGGAGATGTGTATAAGAGACAG-CGTTCATBGCGTAGT TVGGRTAGT |  |  |  |

1.Mihajlovski A, Dore J, Levenez F, Alric M, Brugere JF. Molecular evaluation of the human gut methanogenic archaeal microbiota reveals an age-associated increase of the diversity. Environmental microbiology reports. 2010;2(2):272-80. doi: 10.1111/j.1758-2229.2009.00116.x. PubMed PMID: 23766078.

2.Poulsen M, Schwab C, Jensen BB, Engberg RM, Spang A, Canibe N, et al. Methylotrophic methanogenic Thermoplasmata implicated in reduced methane emissions from bovine rumen. Nature communications. 2013;4:1428. doi: 10.1038/ncomms2432. PubMed PMID: 23385573.

3.Angel R, Matthies D, Conrad R. Activation of methanogenesis in arid biological soil crusts despite the presence of oxygen. PloS one. 2011;6(5):e20453. doi: 10.1371/journal.pone.0020453. PubMed PMID: 21655270; PubMed Central PMCID: PMCPMC3105065.

4.Tymensen LD, McAllister TA. Community structure analysis of methanogens associated with rumen protozoa reveals bias in universal archaeal primers. Appl Environ Microbiol. 2012;78(11):4051-6. doi: 10.1128/AEM.07994-11. PubMed PMID: 22447586; PubMed Central PMCID: PMC3346394.

5.Yu Y, Lee C, Kim J, Hwang S. Group-specific primer and probe sets to detect methanogenic communities using quantitative real-time polymerase chain reaction. Biotechnol Bioeng. 2005;89(6):670-9. doi: 10.1002/bit.20347. PubMed PMID: WOS:000227247700006.

6.Sundberg C, Al-Soud WA, Larsson M, Alm E, Yekta SS, Svensson BH, et al. 454 pyrosequencing analyses of bacterial and archaeal richness in 21 full-scale biogas digesters. FEMS microbiology ecology. 2013;85(3):612-26. doi: 10.1111/1574-6941.12148. PubMed PMID: 23678985.
